# Genome-Wide Analysis of Citrus *R2R3MYB* Genes and Their Spatiotemporal Expression under Stresses and Hormone Treatments

**DOI:** 10.1371/journal.pone.0113971

**Published:** 2014-12-04

**Authors:** Rangjin Xie, Yongjie Li, Shaolan He, Yongqiang Zheng, Shilai Yi, Qiang Lv, Lie Deng

**Affiliations:** Citrus Research Institute, Chinese Academy of Agricultural Science, Southwest University, Chongqing, China; Universidade Federal do Rio Grande do Sul, Brazil

## Abstract

The R2R3MYB proteins represent one of the largest families of transcription factors, which play important roles in plant growth and development. Although genome-wide analysis of this family has been conducted in many species, little is known about *R2R3MYB* genes in citrus, In this study, 101 *R2R3MYB* genes has been identified in the citrus (*Citrus sinesis* and *Citrus clementina*) genomes, which are almost equal to the number of rice. Phylogenetic analysis revealed that they could be subdivided into 21 subgroups. The evolutionary relationships and the intro-exon organizations were also analyzed, revealing strong gene conservation but also the expansions of particular functional genes during the plant evolution. Tissue-specific expression profiles showed that 95 citrus *R2R3MYB* genes were expressed in at least one tissue and the other 6 genes showed very low expression in all tissues tested, suggesting that citrus *R2R3MYB* genes play important roles in the development of all citrus organs. The transcript abundance level analysis during abiotic conditions (NaCl, abscisic acid, jasmonic acid, drought and low temperature) identified a group of *R2R3MYB* genes that responded to one or multiple treatments, which showed a promising for improving citrus adaptation to stresses. Our results provided an essential foundation for the future selection of the citrus *R2R3MYB* genes for cloning and functional dissection with an aim of uncovering their roles in citrus growth and development.

## Introduction

*MYB* gene family is large, functionally diverse and present in all eukaryotes, the proteins encoding by which usually function as transcription factors with MYB binding domain conferring the ability to bind DNA. The MYB domain is consisting of one to four imperfect repeats (R), and each repeat has about 52 amino acid residues [1]. Based on the number of repeat(s) in the MYB domain, MYB proteins are divided into four types: 4RMYB contains four repeats, 3RMYB (R1R2R3MYB) has three consecutive repeats, R2R3MYB possesses two repeats, and the MYB-related type usually, but not always, has a single repeat [2]. Among these four types, R2R3MYB is specific to higher plants and quantitatively predominant in most plants, which is characterized by the presence of a conserved MYB domain and a highly variable C-terminal region [3], [4].

Based on their well conserved DNA-binding domains, genome-wide identification of R2R3MYB members has been conducted in various plants, such as *Arabidopsis* (126 members) [5], *Oryza sativa* (102 members) [5], *Vitis vinifera* (117 members) [6], *Populus trichocarpa* (192 members) [7], *Zea mays* (157 members) [4], *Glycine max* (over 200 members) [3] and *Cucumis sativus* (55) [2]. In *Arabidopsis*, the members of the R2R3MYB family were classified into 25 subgroups [1]. By comparative phylogenetic analysis, Wilkins et al. identified new R2R3MYB subgroups from *Pupulus trichocarpa* [7] that had no representatives in *Arabidopsis*, the same case to some other plant species, suggesting these proteins possess specialized biological functions that have obtained after divergence from the last common ancestor or were lost in *Arabidopsis* or both. The expansion of the R2R3MYB transcription factors in plants is well favor of the observation that numerous R2R3MYB proteins play central roles in plant-specific processes [7].

A growing body of evidence demonstrate that R2R3MYB transcription factors are involved in plant numerous physiological and biochemical processes, such as leaf trichome differentiation [8], secondary wall formation [9], anther and pollen development [10], [11], axillary meristem formation [12], the regulation of secondary metabolism including flavonoids [13], anthocyanin [14] and lignins [15]. Besides that, R2R3MYB family members also take part in plant defense and response to various abiotic and biotic stresses [16]–[19] and have demonstrated roles in regulating plant responses to phytohormonal cues including indole acetic acid [20], abscisic acid [20], [21], gibberellins [22], [23], ethylene, salicylic acid and jasmonic acid [23] and to environmental signals, such as water availability [24], light [25] and nutrient elements [26].

The functions of R2R3MYB genes have been extensively studied in various plant species, which provided us a better understanding of this gene subfamily. However, very little is known about this gene subfamily in citrus. To the best of our knowledge, all available data, thus far, about *R2R3MYB* genes in citrus are related to anthocyanin biosynthesis [27], [28]. Citrus as one of the most important economic crops for its high healthy value is widely grown all over the world. However, its growth and production are severely affected by numerous biotic and abiotic stresses including drought, temperature extremes, salinity and pathogens. Therefore, identification and functional analysis of citrus defense- and stress-related genes may help to elucidate the molecular mechanisms underlining the plant defense and to improve plant stress tolerance.

Recently, two citrus genome sequences including sweet orange (*Citrus sinesis*) and clementine (*Citrus clementina*) were released (http://www.phytozome.net), thereby enabling genome-wide identification and analysis of citrus *R2R3MYB* genes to be conducted. In the present research, *R2R3MYB* genes were isolated based on genomic information available at (http://www.phytozome.net). Phylogenetic and structural analysis was conducted using the citrus *R2R3MYB* genes according to sequence data. Organ specific transcription profiles of *R2R3MYB* genes were constructed for various organs from *Citrus junos* cv. ‘Ziyang’. Furthermore, the temporal expression analysis of *R2R3MYB* genes in response to stresses and hormones was also performed, which helped us to identified the potentially genes that participate in the stress signal transduction pathway in citrus. Additionally, these results, for the first time, provide information upon the relationship between functional divergence and evolution in citrus R2R3MYB subfamily.

## Materials and Methods

### Identification of Citrus *R2R3MYB* Genes

To identify the citrus *R2R3MYB* genes from citrus (sweet orange and clementine) genome (http://www.phytozome.net), a BLASTP search has been performed at the Join Genome Institute (JGI) (http://www.phytozome.net) using the amino acid sequences of *Arabidopsis* R2R3MYBs. All of the sequences having MYB domain were obtained from the citrus genome database. To further confirm the reliability of our results, the functional and structural domains were predicted by PROSITE profiling (http://www.expasy.org/tools/scanprosite/) [29] and SMART analysis (http://smart.embl-heidelberg.de/) [30], respectively. Only the sequences having two repeats (R2 and R3) were selected as the candidates.

In addition, based on the results reported by Stracke et al. [31], the sequences of 126 *Arabidopsis* R2R3MYB proteins were downloaded from the PlantTFDB (http://planttfdb.cbi.pku.edu.cn), respectively.

### Sequence Analysis

In order to analyze the sequence features of the 101 predicted Citrus R2R3MYB proteins, multiple sequence alignment of the MYB domains was performed using ClustalX [32] with default parameters. The deduced amino acid sequences were adjusted manually using BioEdit (version 7.0.0) with default parameters (The National Resource for Biomedical Supercomputing (NRBSC): http://www.nrbsc.org/) [33] with the aim to obtain optimized alignment.

The intron pattern can serve as an independent criterion in support of subgroup designations of phylogenetic analysis. Therefore, intron pattern analysis of *CitMYB* genes was performed. The genomic and cDNA sequences corresponding to each predicted *CitMYB* genes were uploaded based on the results of BLASTP searches in the citrus genome database, and their intron distribution patterns, phases and intro-exon boundaries were analyzed using the GSDS web-based bioinformatics tool (http://gsds.cbi.pku.edu.cn/).

The chromosomal position of genes was provided by the Citrus Genome Database. The distribution of *CitRR3MYB* genes throughout the sweet orange and clementine genomes was drawn manually.

### Phylogenetic Analysis

Based on the aligned sequences of the citrus R2R3MYB proteins, a Neighbor Joining (NJ) tree was constructed using MEGA version 4.0 with a bootstrap of 1000 replicates, aiming to investigate the evolutionary history of the R2R3MYB genes in citrus. In order to predict the functions of the citrus *R2R3MYB* genes, a combined CitMYB (101 members) and AtMYB (126 members) phylogentic tree was created, also using MEGA 4.0 with NJ method and a bootstrap of 1000 replicates.

### Expression Profiling of Citrus *R2R3MYB* Genes

To investigate the expression profiles of *CitMYB* genes in response to abiotic stress and plant hormone, citrus (*Citrus junos* Sieb. cv. ‘Ziyang’) seeds were peeled, and germinated on moist filter paper in a dark chamber with 28°C and 100% relative humidity for 6 days. The germinated seeds were sown into nutritive soil and then placed in an illuminated chamber (28°C, 80% relative humidity and 350 µmol m^−2 ^s^−1^ light intensity) throughout the experiment, which were irrigated with water every 3 days. When seedlings were at two-true-leaf stage, six treatments were treated, respectively: 200 mM NaCl, dehydrate, 0°C low temperature, 150 µM abscisic acid (ABA) and 200 µM jasmonic acid (MeJA). Roots and leaves used for RNA extraction were harvested at 0, 1 and 6 h after six treatments, of which the materials collected at 0 h served as the control. The flower, fruitlet, root and leaves of mature were collected for tissue specific expression analysis. All the samples were stored at −80°C until used.

Total RNA was isolated from different tissues using RNApre pure plant Kit (TIANGEN, China) according to the manufacturer’s instructions. Two µg total DNA-free RNA was used to synthesized first strand cDNA with PrimeScript 1^st^ Strand cDNA Synthesis Kit (TaKaRa, Japan).

Real-time PCR using SAND gene [34] as normalize was performed according to the manufacturer’s specifications (SYBR PrimeScrip RT-PCR Kit; TaKaRa, Dalian, Liaoning, P. R. China). SYBR Green PCR was carried out using the iCycler iQ5 real-time detection system (Bio-Rad) for 30 s at 95°C, followed by 40 cycles of 10 s at 95°C, 30 s at 60°C, and 30 s at 72°C, with a final step at 72°C for 1 min. Each expression profile was independently verified in 3 replicate experiments performed under indentical conditions. Each relative level of gene expression was calculated by the 2^–ΔΔCt^ method [35]. The PCR primers were designed outside the conserved region to produce amplification products with 130–200 bp. All primer sequences were detailedly listed in Table S1.

The data obtained were statistically analysed using DPS Version 7.55 (http://www.chinadps.net; Zhejiang University, Hangzhou, P. R. China). One-way ANOVA and Duncan’s new multiple-range test were used to determine significant differences in mean values among materials at 0 h, 1 h and 6 h and P≤0.05 was regarded as significant.

## Results

### Identification of the Citrus *R2R3MYB* Genes in Citrus Genome

**Figure 1:**
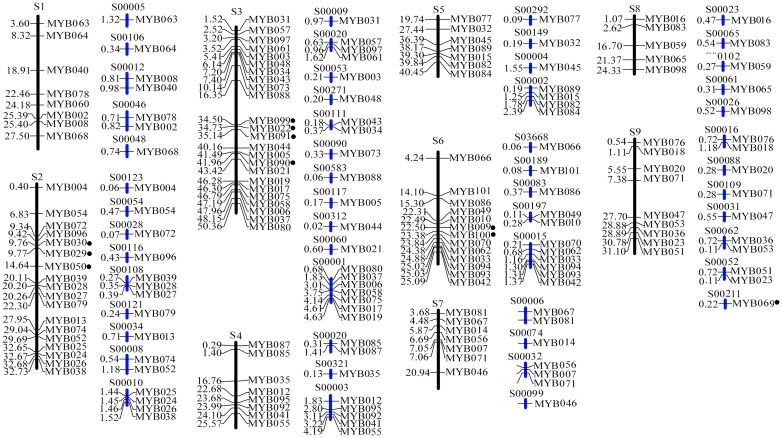
Chromosomal locations of 101 *CitMYB* genes. The scaffolds (S) of sweet orange and clementina were indicated by blue and black columns. The numbers on the left of scaffolds show the relative position of *CitMYB* genes. The scale is in megabases (Mb). The species-specific *CitMYB* genes are highlighted by solid black dot.

One hundred and twenty-six *Arabidopsis* R2R3MYB proteins were used as a query to search against the citrus (sweet orange and clementine) genomes at the Join Genome Institute (JGI) (http://www.phytozome.net) with BLASTP program. A total of 128 MYB related sequences with MYB domain were identified. To confirm putative *R2R3MYB* genes, PROSITE and SMART were employed to search for the amino acid sequences of all 128 proteins, and 101 typical *R2R3MYB* genes (named *CitMYB001* to *CitRMYB101*) were confirmed. These 101 *CitMYB* genes were used to further analysis (Table 1), of which 10 genes including *CitMYB013, 014, 028, 030, 033, 060, 075, 092, 093* and *097* were specifically present in the clementine genome, one (*CitMYB099*) specifically in the sweet orange genome and 90 in both genomes (Fig. 1). In addition, comparative analysis showed that the genome distribution of *R2R3MYB* genes was highly conserved between the sweet orange and clementine.

**Table 1:** *R2R3MYB* genes in Citrus.

	Genename	Locusname	ORF(bp)	Exonnumber	Exonlength (pb)					Predicted protein		
					E1	E2	E3	E4	E5	Length(aa)	PI	Mass(Da)
C1	CitMYB001	Ciclev10026498m	624	3	136	130	358	-	-	207	7.69	23426.1
	CitMYB002	Ciclev10009521m	605	3	136	130	343	-	-	202	8.96	23119
	CitMYB003	Ciclev10022303m	612	3	160	130	322	-	-	203	8.42	23272.9
C2	CitMYB004	Ciclev10015648m	1125	1	1125	-	-	-	-	374	5.86	41334.5
	CitMYB005	Ciclev10023255m	870	2	302	568	-	-	-	395	8.18	44020.9
	CitMYB006	Ciclev10021571m	843	2	133	710	-	-	-	280	8.66	31525.8
	CitMYB007	Ciclev10027480m	558	5	4	132	14	110	298	186	9.82	21464.6
C3	CitMYB008	Ciclev10009286m	753	3	145	130	478	-	-	250	9.08	28439
	CitMYB009	Ciclev10013455m	723	4	118	130	133	342	-	240	7.11	27686.1
	CitMYB010	Ciclev10013828m	651	4	43	130	46	432	-	216	8.38	24348.7
C4	CitMYB011	Ciclev10017556m	675	4	136	130	338	71	-	224	8.65	25465.4
	CitMYB012	Ciclev10032713m	654	3	118	130	406	-	-	217	8.24	25368.6
C5	CitMYB013	Ciclev10017654m	645	3	130	130	385	-	-	214	8.82	24178.3
	CitMYB014	Ciclev10026112m	951	3	133	130	688	-	-	316	8.97	35167.1
	CitMYB015	Ciclev10002068m	867	3	133	130	604	-	-	288	9.12	33146.1
	CitMYB016	Ciclev10028908m	924	2	263	661	-	-	-	307	9.2	33954.4
C6	CitMYB017	Ciclev10020336m	1254	1	1254	-	-	-	-	417	4.76	46726.9
	CitMYB018	Ciclev10005376m	1014	3	133	130	751	-	-	337	5.42	37645
	CitMYB019	Ciclev10023756m	1425	3	133	130	1162	-	-	474	4.72	52890.8
	CitMYB020	Ciclev10005629m	810	3	133	130	547	-	-	269	6.22	30095.1
	CitMYB021	Ciclev10021699m	804	3	133	130	541	-	-	267	5.56	29988.4
	CitMYB022	Ciclev10022057m	696	3	133	130	433			231	9.19	26762.4
	CitMYB023	Ciclev10005102m	1206	4	4	171	130	901	-	401	5.09	44840.7
C7	CitMYB024	Ciclev10018064m	735	3	133	130	472	-	-			
	CitMYB025	Ciclev10017679m	372	3	133	130	109	-	-	124	10.39	14891.1
	CitMYB026	Ciclev10018064m	879	4	113	20	130	616	-	292	6.02	33227.1
	CitMYB027	Ciclev10016303m	786	3	133	130	523	-	-	261	6.32	29821.3
	CitMYB028	Ciclev10017677m	732	3	133	130	469	-	-	243	6.05	28354
	CitMYB029	Ciclev10017435m	654	3	133	130	391	-	-	217	5.77	25020.6
	CitMYB030	Ciclev10018311m	657	3	133	130	394	-	-	218	5.8	25443
	CitMYB031	Ciclev10021161m	972	3	133	130	709	-	-	323	5.62	36684.7
	CitMYB032	Ciclev10003251m	960	2	266	694	-	-	-	319	5.82	36135.3
C8	CitMYB033	Ciclev10012152m	1011	3	133	130	748	-	-	336	6.02	37078.3
	CitMYB034	Ciclev10020967m	1032	3	133	130	769	-	-	343	6.31	38128.2
	CitMYB035	Ciclev10031946m	1065	3	133	130	802	-	-	354	6.27	39252.1
	CitMYB036	Ciclev10006455m	882	3	139	130	613	-	-	294	8.31	32834.1
	CitMYB037	Ciclev10021268m	939	3	133	130	676	-	-	312	6.66	34480.7
C9	CitMYB038	Ciclev10015729m	1080	3	133	130	817	-	-	359	6.07	40956.6
	CitMYB039	Ciclev10017668m	1059	3	133	130	796	-	-	352	6.45	39479.3
	CitMYB040	Ciclev10009050m	894	3	133	130	465	166	-	297	8.91	33303.7
C10	CitMYB041	Ciclev10033327m	1104	3	133	130	841	-	-	367	7.66	40397.6
	CitMYB042	Ciclev10011948m	1155	3	133	130	890	-	-	384	6.19	42213.7
	CitMYB043	Ciclev10023440m	1092	3	133	130	766	-	-	342	8.03	38369.6
C11	CitMYB044	Ciclev10020765m	1089	3	266	786	37	-	-	362	5.85	40346
	CitMYB045	Ciclev10003958m	2094	5	133	130	917	130	784	348	5.86	38774.4
	CitMYB046	Ciclev10026023m	1008	3	133	130	745	-	-	335	6.59	37120.7
C12	CitMYB047	Ciclev10005666m	786	2	263	523	-	-	-	261	5.93	29641
	CitMYB048	Ciclev10021695m	804	2	263	541	-	-	-	267	5.14	30208.8
	CitMYB049	Ciclev10012265m	936	3	133	130	673	-	-	311	5.1	35536.8
	CitMYB050	Ciclev10017764m	798	3	133	130	535	-	-	265	5.99	30674.3
C13	CitMYB051	Ciclev10006752m	1035	3	130	130	775	-	-	344	6.82	38840.9
	CitMYB052	Ciclev10018009m	1017	2	130	887	-	-	-	338	6.11	38009.8
	CitMYB053	Ciclev10006897m	942	3	148	130	664	-	-	313	6.46	35168.7
C14	CitMYB054	Ciclev10017658m	951	2	290	661	-	-	-	316	5.74	35313.3
	CitMYB055	Ciclev10033941m	999	2	284	715	-	-	-	332	5.15	37238.6
	CitMYB056	Ciclev10027201m	1140	3	133	130	877	-	-	379	6.18	42172.4
C15	CitMYB057	Ciclev10024338m	1191	3	133	130	928	-	-	369	5.62	44372.9
	CitMYB058	Ciclev10020144m	1341	3	133	130	1078	-	-	446	7.62	49473.8
	CitMYB059	Ciclev10028398m	1377	2	347	1030	-	-	-	458	6.23	50885.6
C16	CitMYB060	Ciclev10008921m	1944	5	133	130	842	130	709	323	7.12	35905.4
	CitMYB061	Ciclev10023930m	1155	3	133	130	892	-	-	384	5.57	43026.1
	CitMYB062	Ciclev10013340m	1005	3	133	130	742	-	-	334	8.12	36860.1
	CitMYB063	Ciclev10010621m	585	3	136	130	319	-	-	195	9.03	21576.2
	CitMYB064	Ciclev10009336m	717	4	136	130	185	266	-	238	4.97	26148.9
	CitMYB065	Ciclev10028804m	1011	3	136	130	745	-	-	336	6.75	38121.4
	CitMYB066	Ciclev10012151m	1011	4	136	130	683	62	-	336	6.1	37284.4
	CitMYB067	Ciclev10027178m	1065	3	136	130	799	-	-	354	5.85	39667.4
	CitMYB068	Ciclev10010364m	1104	3	136	130	838	-	-	367	6.02	41152.6
C17	CitMYB069	Orange1.1g044161m	690	4	115	130	101	344	-	229	6.24	26695.7
	CitMYB070	Ciclev10013466m	753	3	130	130	493	-	-	251	8.91	28797.2
	CitMYB071	Ciclev10006914m	783	3	142	130	511	-	-	260	9.13	30625.4
	CitMYB072	Ciclev10016820m	585	3	136	130	319	-	-	194	5.85	22373
	CitMYB073	Ciclev10021157m	975	3	160	130	685	-	-	324	5.99	37010.6
	CitMYB074	Ciclev10015986m	951	3	175	130	646	-	-	316	5.6	36153.2
	CitMYB075	Ciclev10021479m	870	3	169	130	571	-	-	289	5.99	33048.3
	CitMYB076	Ciclev10005387m	1005	3	181	130	694	-	-	334	7	37578.9
C18	CitMYB077	Ciclev10000756m	1656	3	357	994	305	-	-	474	5.76	51822.9
	CitMYB078	Ciclev10010620m	12920	3	345	778	167	-	-	429	8.67	47002.7
	CitMYB079	Ciclev10014958m	1536	3	312	928	296	-	-	511	5.65	55342.1
	CitMYB080	Ciclev10021345m	909	3	188	250	471	-	-	302	5.83	34358.7
C19	CitMYB081	Ciclev10026107m	954	1	954	-	-	-	-	317	8.93	34170.2
	CitMYB082	Ciclev10001979m	909	1	909	-	-	-	-	302	8.46	32948.9
	CitMYB083	Ciclev10029124m	759	1	759	-	-	-	-	252	5.8	28352.4
	CitMYB084	Ciclev10002239m	765	1	765	-	-	-	-	254	8.75	28053.9
	CitMYB085	Ciclev10032317m	858	1	858	-	-	-	-	258	6.13	31388.9
	CitMYB086	Ciclev10012003m	1110	2	346	764	-	-	-	369	5.64	40172.9
	CitMYB087	Ciclev10031463m	1389	2	316	1073	-	-	-	462	5.51	49989
	CitMYB088	Ciclev10021859m	750	3	115	456	179	-	-	249	9.16	29204.3
	CitMYB089	Ciclev10001803m	996	2	169	827	-	-	-	331	9.33	37472.3
	CitMYB090	Ciclev10020631m	1134	3	436	408	290	-	-	377	7.7	41808.5
	CitMYB091	Ciclev10023285m	738	1	738	-	-	-	-	245	10.04	28366.1
	CitMYB092	Ciclev10031944m	1068	4	6	202	540	320	-	355	6.89	40016.4
	CitMYB093	Ciclev10011967m	1140	3	460	543	137	-	-	379	7.65	41988.6
	CitMYB094	Ciclev10011967m	1188	3	460	393	335	-	-	395	8.81	44020.9
C20	CitMYB095	Ciclev10031405m	1431	12	81	47	57	73	56	444	6.66	49952.5
	CitMYB096	Ciclev10017526m	1386	3	629	127	630	-	-	461	7.8	52300.4
	CitMYB097	Ciclev10024427m	1296	3	584	127	585	-	-	431	6.31	49404.2
	CitMYB098	Ciclev10030254m	1377	3	518	127	732	-	-	458	7.31	52083.3
C21	CitMYB099	Ciclev10018691m	2982	4	177	1722	282	801	-	993	5.29	111366
	CitMYB100	Ciclev10011915m	1182	1	1182	-	-	-	-	393	9.24	44703.8
	CitMYB101	Ciclev10012089m	1053	1	1053	-	-	-	-	350	9.25	40422.1

### Conserved Residues in the MYB Domain

**Figure 2:**
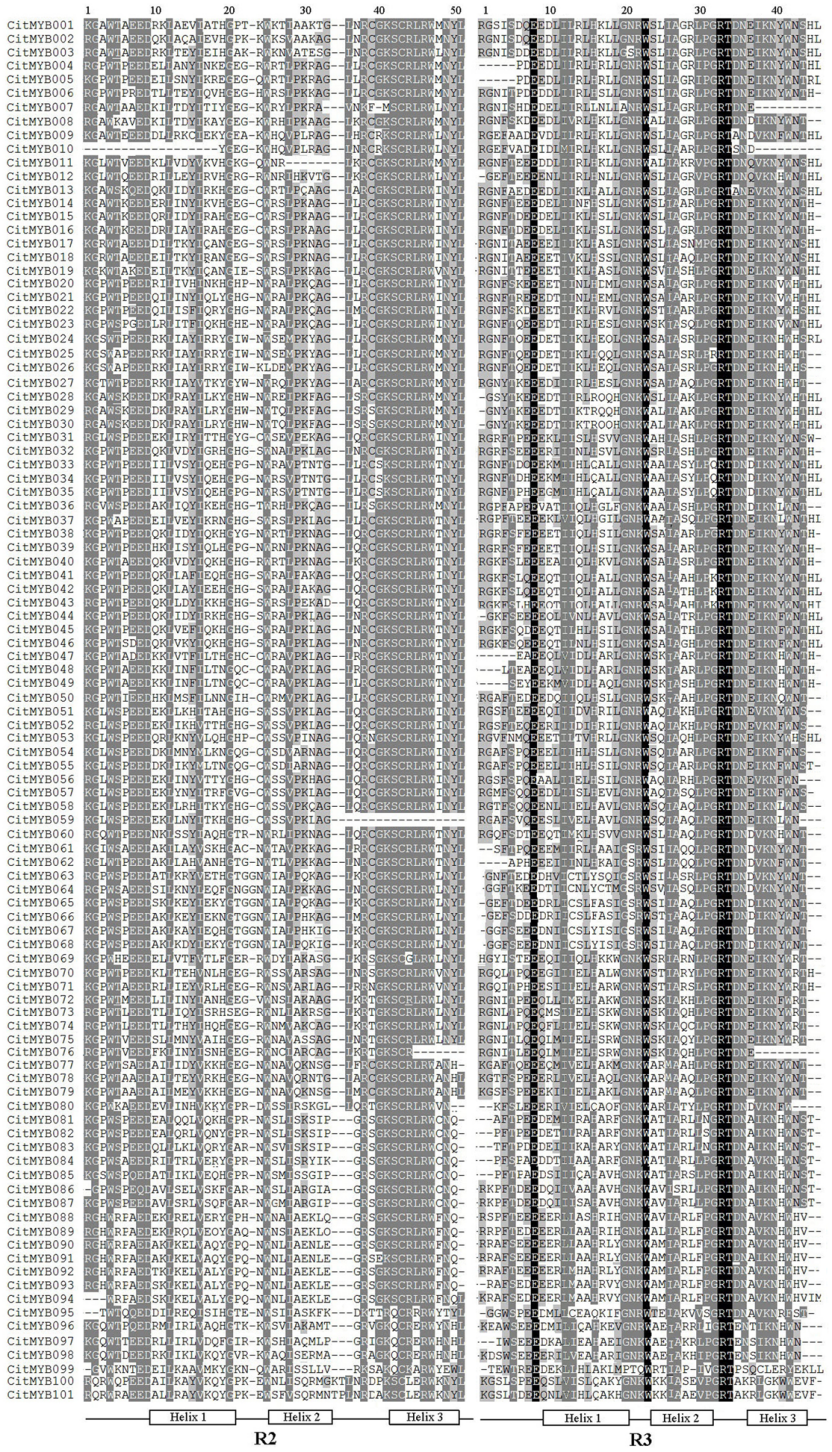
ClustalW amino acid sequence alignment of 101 citrus R2R3-MYB domains. The shading of the alignment represents different degrees of conservation among sequences; the dark shading indicates identical residues, the light shading indicates conservative changes. The positions of the three a-helices that form each MYB repeat are marked as Helix 1 to Helix 3.

To gain insight into the citrus R2R3MYB binding domains, amino acid sequence alignment was conducted to examine how well conserved the R2 and R3 repeats were in the R2R3MYB proteins within each residue position. As shown in Fig. 2, the basic regions of citrus R2R3MYB domains contained, on average, approximate 100 basic residues, with rare frequency of insertion or deletion. By contrast, the region outside the MYB binding domain was poorly conserved in terms of length as well as amino acid composition. Based on previous reports, the R2 and R3 repeats possessed characteristic amino acids, including a series of evenly distributed and highly conserved Trp (W) residues [2], [4]. Within the 101 citrus R2R3MYB proteins, 97 of their R2 repeat sequences contained three tryptophan residues, which located at 4, 25 and 47, forming a hydrophobic core and serve as landmarks in plant MYB binding domain. However, in the R3 repeat, the first tryptophan residue (located at 4) of most members was replaced by phenylalanine. The second (located at 23) and third tryptophan residues (located at 42) were well conserved in almost all citrus R2R3MYB proteins, especially the second one which exist in all members.

### Phylogenetic Analysis of the Citrus R2R3MYB Family

**Figure 3:**
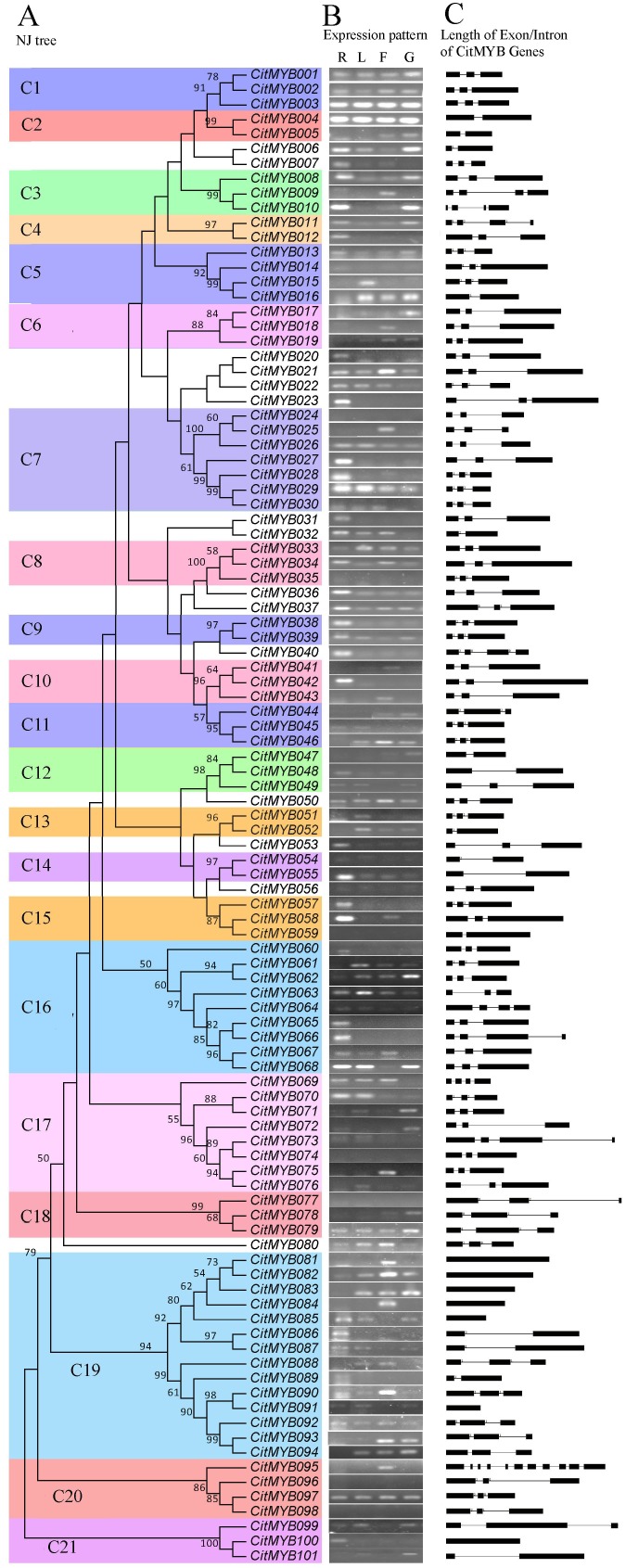
Phylogenetic relationships, intron pattern, expression pattern, and subgroup designations in R2R3MYB proteins from citrus. A, The neighbor-joining (NJ) tree based on the complete protein sequences of 101 CitMYBs and the bootstrap values less than 50 are not shown in NJ tree. The tree shows the 21 phylogenetic subgroups (C1–C21) marked with colored backgrounds. Twelve proteins did not fit well into clusters. B, The gene structure is presented by exon (black boxes) and intron (black line between the black boxes). The number indicated the phases of corresponding introns. C, The expression patterns of 101 CitMYB genes in different organs. The letter R above the column of expression data refers to root, L refers to leaf, F refers to flower and G refers to fruitlet.

The phylogenetic relationship between the citrus R2R3MYB proteins has been examined by multiple sequence alignment of their whole protein sequences using the NJ method with bootstrap analysis (1,000 replicates). The 101 members of the citrus R2R3MYB family were subdivided into 21 subgroups, designated C1 to C21, according to clades with at least 50% bootstrap support (Fig. 3). Additionally, our results also showed that the phylogenetic trees established with MYB binding domains and whole protein sequences, respectively, were composed of nearly identical subgroups, despite the classification of only a few member varied (Fig. 3; Fig. S1). This result indicated that the phylogenetic relationship between citrus R2R3MYBs based on the whole protein sequence was mainly decided by MYB binding domains, and those citrus R2R3MYBs within the same subgroup may bind to the same MYB recognition sequence, while the regulatory functions of which probably were divergent because of the dramatic divergence of their C-terminal regions that is the main transcriptional activation domain responsible for functional activity or/and specificity [36]. Thus, the phylogenetic tree built, in this study, with C-terminal regions of citrus R2R3MYBs seems more appropriate for revealing the similarity and divergence of regulatory function of the corresponding proteins.

**Figure 4:**
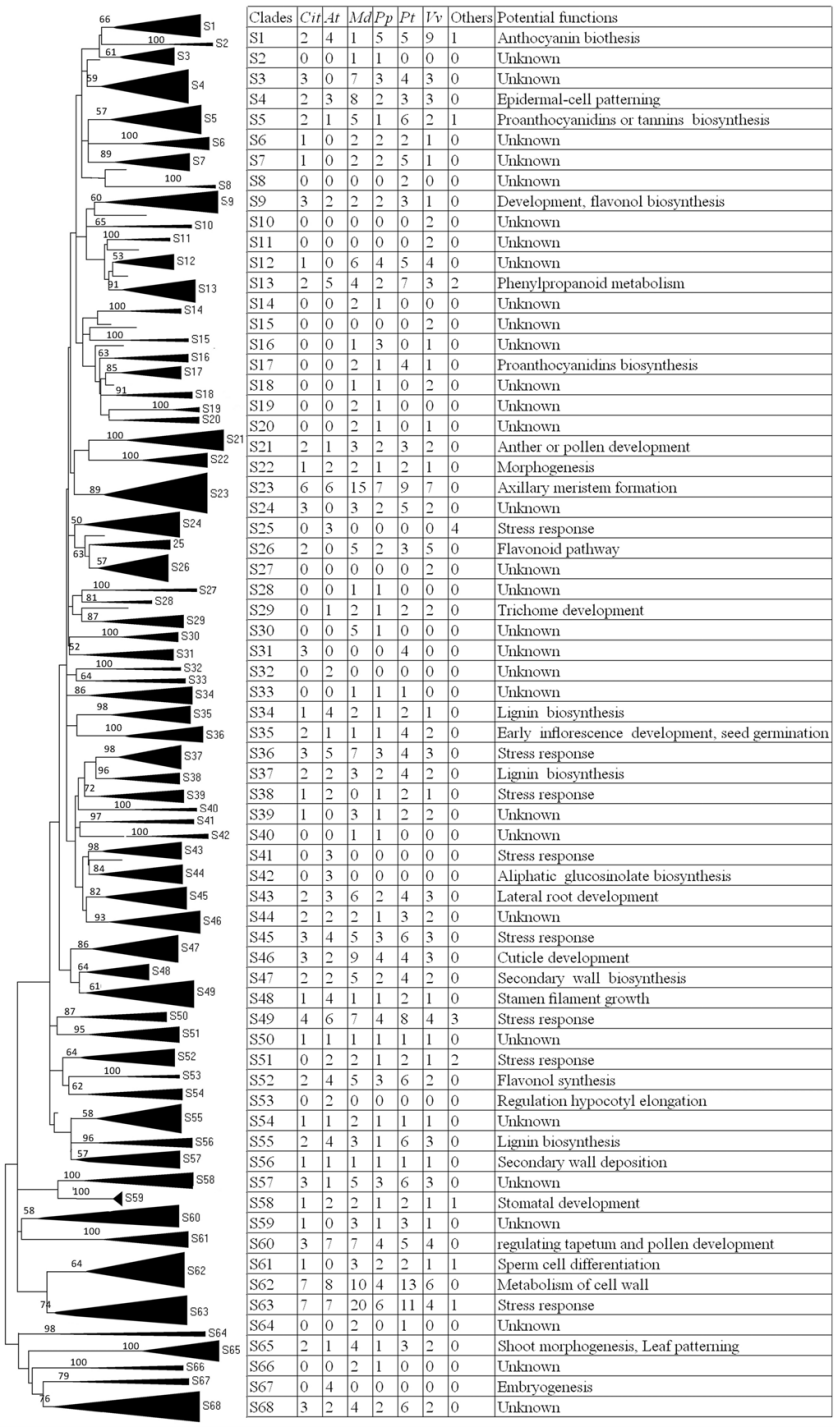
Neighbor-joining (NJ) tree of the R2R3MYB proteins from citrus (*Cit*), Arabidopsis (*At*), apple (*Md*), grape (*Vv*), peach (*Pp*), populus (*Pt*) and other plant species. The NJ tree was built with 101 R2R3MYB proteins from citrus, 126 from *Arabidopsis*, 214 from apple, 126 from grape, 192 from populus, 110 from peach and 25 well characterized R2R3MYB proteins from other plant species. The proteins are clustered into 68 subgroups (triangles), designated as S1 to S68. Bootstrap values less than 50 are not shown in the NJ tree. Eleven proteins did not fit well into clusters. The table on the left contained the information that explained NJ tree.

To better understand the functional clades with the citrus *R2R3MYB* genes, an unrooted NJ phylogenetic tree using bootstrap analysis (1000 replicates) was established by alignments of the whole protein sequences of R2R3MYBs from citrus (101), *Arabidopsis* (126), apple (214), grape (126), peach (110) and populus (192) as well as 25 well characterized R2R3MYBs of other plant species such as pear, Chrysanthemum, wheat, tobacco, rice and *Leucaena leucocephala* (Fig. 4). The resulting tree generated 68 subgroups (sequentially termed as S1 to S68) with at least 50% bootstrap support, which was similar to the results previously reported [2], [4]. As shown in Fig. 4, 41 out of 68 subgroups were shared with citrus and other plant species. This indicated that most *R2R3MYB* genes in these species highly conserved during plant evolution. Meanwhile, ten species-specific subgroups such as S8, 10, 11, 15 and 32 were observed, indicating that these *R2R3MYB* genes may have evolved or been lost in a plant species following divergence. Interestingly, of these ten species-specific subgroups, none contained MYB members from citrus, which suggested that these genes may possess specialized roles in other plant species, while are probably dispensable in citrus. As expected, two CitMYBs (CitMYB007, CitMYB008 and CitMYB022) were not contained in any one of 68 subgroups, the functions of which are worth to detailedly elucidate in future.

### Intro-exon Structure of the Citrus R2R3MYB Family

**Figure 5:**
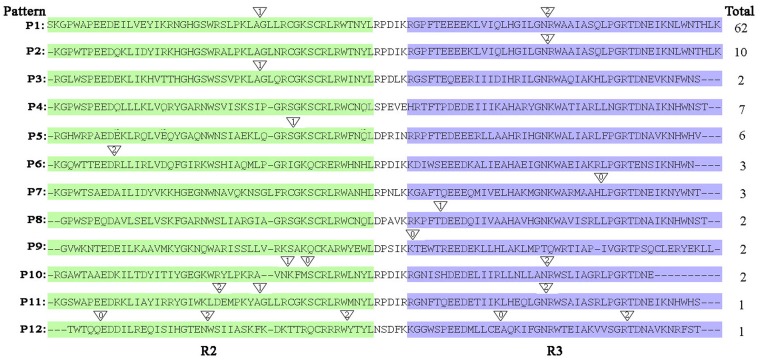
Schematic of the intron distribution patterns within the MYB domains of 101 CitMYBs. Alignment of the MYB domains is representative of 12 intron patterns, named from P1 to Pl2. The locations of introns are indicated by white triangles. The number within each triangle indicates the splicing phases: 0 refers to phase 0; 1 refers to phase 1; and 2 refers to phase 2. The number of CitMYB proteins with each pattern is shown on the right.

Among 101 citrus R2R3MYBs, up to 97 of them possessed at least 1 intron in the R2 and R3 domains. According to their relative positions and phases, all genes could be grouped into 12 patterns (P1–12) (Fig. 5). By contrast, outside the MYB domain, all but 20 of the 101 citrus R2R3MYBs lacked introns.

Pattern P1–3, composed of one or two intron (s) distributed at two highly conserved specific positions, accounting for approximately 73% of CitMYBs. Patterns P5-P12 contained 1 to 5 introns at varying positions in the R2 or R3 domain, were observed in less than 20% of the 101 CitMYBs. In addition, approximately 7% of CitMYBs have no intron in their R2 or R3 domain, forming the third main intron pattern. Intron phases in regard to codons were also investigated in this study. Fig. 5 showed that in the major splicing patterns P1, P2 and P3, the introns phases were 1 and/or 2, respectively, where the phase at the same position of the R2 domain was 1, and that at the R3 was 2.

### Organ-Specific Expression Analysis

The expression profiles of citrus R2R3MYB gene family were also analyzed using root, leaf, flower and fruitlet. The results revealed that most of CitMYBs could express in at least one organ. However, few *CitMYB* genes, including *CitMYB024, 035, 059, 074, 077, 096*, did not show expression signals (Fig. 3), suggesting that these genes may be pseudogenes, or may be expressed at specific development or under special conditions. The rest showed remarkably variation in transcript abundance, characterized by high level of transcript abundance in one or some organs and low transcript abundance in others. The wide expression of *CitMYB* genes in different organs indicated that they may play important roles in the development of all citrus organs.

As shown in Fig. 3, 20 out of the 101 *CitMYB* genes (*CitMYB001–004, 013, 021, 026, 029, 033, 036, 037, 039, 050, 055, 062, 063, 079, 082, 092, 097*) were expressed in all 4 organs, although the transcript abundance of some genes was very low, indicating they may play important regulatory roles in growth and development of citrus. There were 18 *CitMYB* genes with preferential expression in root, including *CitMYB023*, *027–028, 031, 036, 038, 040 042, 048, 053, 055, 057–058, 060, 065–066, 086* and *100*. Six *CitMYB* genes (*CitMYB015*, *051–052*, *061*, *063*, *076*) were preferentially expressed in leaf. Additionally, ten and four *CitMYB* genes showed preferential expression in flower and fruitlet, respectively, indicating they may involve in reproductive development. It is generally accepted that the similar expression pattern of closely clustering members implied the function redundancy, for example the members in subgroup C1, by contrast the member with different expression pattern may play the same role in different stage during citrus development.

### Expression Profiles of the Citrus *R2R3MYB* Genes in Response to Abiotic Stresses and Hormones

**Figure 6:**
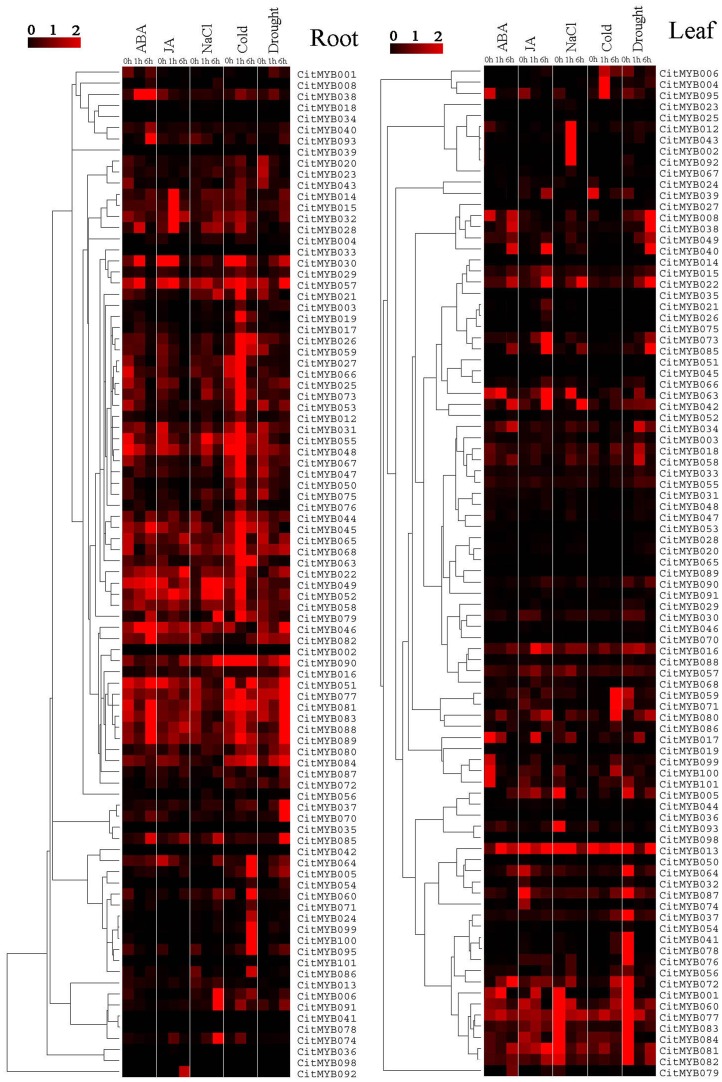
Heatmap of expression profiles of 101 *CitMYB* genes in leaf (left) and root (right) under different treatments. Transcript abundance of genes is indicated in *color*, with black representing low level and *red* representing high level. Transcript abundance was evaluated by real-time PCR. Heatmap was constructed with sofeware TreeView 1.60.

In this study, the transcript abundances of the 101 citrus *R2R3MYB* genes in leaf and root of citrus seedling at two-true-leaf stage were investigated under cold (0°C), drought, NaCl (200 mM), ABA (150 µM) and MeJA (200 µM) treatments. The results showed that almost all genes responded to at least one treatment in root and/or leaf (Fig. 6). Numerous *CitMYB* genes could be positively regulated by one treatment, while being negatively regulated by others. For instance, the *CitMYB038* gene was up-regulated by NaCl, ABA and draught, while down-regulated by MeJA. There were a few genes, for example *CitMYB085*, which could be induced by all treatments, suggesting it is a pleiotropic regulator. Interestingly, none of 101 *CitMYB* genes has been found to be repressed by all treatments. Several genes only responded to a single treatment in leaf and/or root. The expression of ten *CitMYB* genes including *CitMYB007, 009–011, 061–062, 069, 094, 096–097*, was very low under all treatments, especially given that *CitMYB096* gene also showed no expression signals in different organs, further indicating it is a pseudogene. As shown in Fig. 6, the expression patterns of most *CitMYB* genes in leaf were significantly different from that in root. For instance, *CitMYB037* in leaf was repressed by NaCl and drought and induced by ABA, MeJA and cold, while in root was induced by all treatments, suggesting the mechanism of this gene in response to NaCl and drought was different in these two organs.

## Discussion

### Characterization of the Citrus R2R3MYB Family

In this study, 101 citrus *R2R3MYB* genes were identified and detailedly characterized. The size of the R2R3family in citrus was smaller than that of *Arabidopsis* (126) [5], populus (197) [7], grape (117) [6], maize (157) [4] and soybean (244) [3], was almost equal to that of rice (102) [5], and was larger than that of cucumber (55) [2], suggesting the *R2R3MYB* gene family in citrus had shrink compared to Arabidopsis, polar, grape and soybean, but expanded compared to cucumber. the number of *R2R3MYB* genes in our study may be particularly true, considering that the total gene number predicted in *Citrus Sinensis* or *Citrus clementina* (24533) is even lower than that in Arabidopsis (26819). However, our data showed that species-specific R2R3MYB members were present in sweet orange or clementina, which let us undoubtedly believe that some new R2R3MYB members were contained in other citrus species such as mandarin (*Citrus reticulata*), sour orange (*Citrus aurantium*), pomelo (*Citrus grandis*), lemon (*Citrus limon*), citron (*Citrus medica*). By this reasoning, the number of the *R2R3MYB* genes in citrus probably is far more than 101, which remain to be further validated.

The MYB binding domains were hightly conserved in 101 citrus R2R3MYB proteins and most of them possessed characteristic amino acids, which were in line with those from *Cucumber* [2], *Populus* [7], *Arabidopsis* [31] and *Triticum* [37]. In addition, our data showed that the splicing phases and patterns of citrus *R2R3MYB* genes were highly conserved during the evolution, which were also observed in the MYB gene families of cucumber [2], soybean [3], maize [4], rice and *Arabidopsis* [5].

Phylogenic Relationship and Function of Citrus R2R3MYB Family Genes.

It is well known that R2R3MYB proteins are involved in a range of different physiological processes, including the response to various stress conditions, secondary metabolism, cell shape and hormone responses. In spite of their importance and large number, very little information has been presented on citrus. Generally, the functions of a gene could be preliminarily predicated through phylogenetic analysis, because the genes grouping into a clade had similar functions and gene structure, and were considered to evolve from a recent common ancestor. By phylogenetic analysis, our results revealed that most subgroups contained the R2R3MYB members from *Arabidopsis*, citrus, apple, grape, peach, populus and other plant species, indicating their functions were highly conserved during plant evolution. Thus, it is entirely feasible that we could predict the functions of the citrus R2R3MYBs according to their phylogenetic relationship with well characterized R2R3MYBs from other plant species.

According to our analysis, Subgroup 1 contained 27 plant R2R3MYBs, including 2 CitMYBs, MdMYB10, PyMYB10, AtMYB75 and NtAn2, implicated in the regulation of anthocyanin biosynthesis [14], [38], [39], [40]. Subgroup 9 was composed of 13 R2R3MYBs from *Arabidopsis* (2 members), citrus (3 members), apple (2 members), peach (2), populus (3) and grape (1 well-characterized member i.e. VvMYBF1) with potential function in the control flavonol biosynthesis [41], [42], the similar case to subgroup 13, 26 and 52. Within subgroup 13, the members of LIMYB1, CmMYB1, AtMYB4 and AtMYB7 act as repressors to negatively regulate phenylpropanoid biosynthesis [43]–[46]. For instance, AtMYB7 negatively regulates flavonol biosynthesis by repressing the genes encoding dihydroflavonol reductase (DFR) and UDP sugar glycosyltransferase (UGT) [43]; while, LIMYB1 represses lignin biosynthesis by down-regulation of the genes encoding phenylalanin ammo-nialyase (PAL), cinnamate 4-hydroxylase (C4H), 4-Coumarate-coenzymeA ligase (4CL). Du et al. [4] reported that all these genes contain C2 motifs, which were well known to participate in repression of phenylpropanoid biosynthesis. By alignment of protein sequences of CitMYB014, CitMYB015 and CitMYB016, we also found that all of them contained C2 motifs (pdLNLEL[R/S]I[G/S])), further demonstrating their potential function in repressing phenylpropanoid biosynthesis. This remains to be elucidated in the future. In addition, CitMYB007 was clustered with AtMYB123 into a subgroup (bootstrap less than 50%), which was associated with proanthocyanin synthesis [43].

It is well known that plant R2R3MYBs widely take part in the control of plant development. Subgroup 43 contained 20 R2R3MYBs, 2 members from citrus, 3 from *Arabidopsis*, 6 from apple, 2 from peach, 4 from populus and 3 from grape, of which AtMYB93, to date, was well elucidated to negatively regulate lateral root development as an interaction partner of the lateral-root-promoting ARABIDILLO proteins [47]. Subgroup 23 consisted of the members that involved in axillary meristems [1]. In subgroup 21, two members from citrus was grouped with AtMYB35 which was involve in pollen development [48]. The CitMYB077, 078 and 079 in subgroup 60 also seem likely to regulate pollen development [11]. However, whether the abovementioned CitMYBs also have similar functions in the control of plant development remain to further demonstrate. Another well-known role of R2R3MYBs is the regulation of cell fate. For example, subgroup4 and 61 clustered with several R2R3MYB proteins which potentially function in the determination of sperm cell differentiation, cell shape and trichome branching [49]–[51].

Recently, accumulating data demonstrated that numerous R2R3MYBs were widely involved into plant adaptation and tolerance to biotic and abiotic stresses. For example, Subgroup 45 consists of 24 R2R3MYBs, including AtMYB41 and AtMYB102, implicated in regulating the resistance to draught and insect [52], [53]. Another example was provided by subgroup 63, which consisted of 56 members, several of them were involved into abiotic and biotic stresses such as disease, salt, draught [54]–[57]. In addition, the citrus R2R3MYB members in subgroup 5, 36, 38, 49 may possess the functions in stress resistance and those in subgroup 22, 34, 37, 46, 47, 48, 55, 58, 62 and 65 maybe play roles in morphogenesis, lignin biosynthesis, cuticle development, secondary wall biosynthesis and stomatal development. Surely, the functions of many subgroups such subgroup 3, 6, 7 couldn’t be predicted due to the absence of well characterized MYB members in them.

### Expression Analysis of *CitMYB* Genes in Response to Abiotic Conditions

A large number of R2R3MYB proteins from different plant species have been characterized by genetic analysis and have been found to play important roles in various abiotic and biotic stresses [1], [16], [53]–[57]. However, no information, to date, is available about citrus *R2R3MYB* gene involved into abiotic and biotic stresses. In general, we could preliminarily predict the biological functions of a gene by its expression patterns. For this reason, the expression patterns of the 101 *CitMYB* genes were investigated under cold, drought, NaCl, ABA and MeJA treatments. The results indicated that most *CitMYB* genes could be induced by at least one treatment, some of them responded to multiple treatments such as *CitMYB022, CitMYB080* and *CitMYB085*. These *CitMYB* genes show a promise for improving citrus adaptation to stresses, especially the *CitMYB* genes that responded to multiple treatments, since plants often undergo multiple stresses concurrently. Additionally, some genes showed opposing expression patterns under different stress conditions, such as *CitMYB016*, *CitMYB017* and *CitMYB030*, which indicated that they played a major role in the plant response to abiotic conditions and involved in communication between different signal transduction pathways [2].

## Supporting Information

Figure S1 NJ phylogenetic tree of the 101 CitMYB members on the basis of the MYB domain. The bootstrap value less than 50 are not shown in the phylogenetic tree.
(TIF)

Figure S2 Phylogenetic relationships and subgroup designations in R2R3MYB proteins in *Arabidopsis*, citrus, apple, peach, populus, grape and other plants.
(TIF)

Table S1 Specific primers of 101 citrus R2R3MYB genes used for real-time PCR in this study. The primer based on *SAND* gene was used as normalizer.
(DOC)

Table S2 The relative expression data of *CitMYB* genes under different stresses and hormone treatments.
(XLSX)
