# Supplementary figures and images for: Genome-Wide Analysis of Citrus R2R3MYB Genes and Their Spatiotemporal Expression under Stresses and Hormone Treatments
Source: PLoS One. 2014 Dec 4;9(12):e113971. doi: 10.1371/journal.pone.0113971 (PMC4256393; doi:10.1371/journal.pone.0113971)

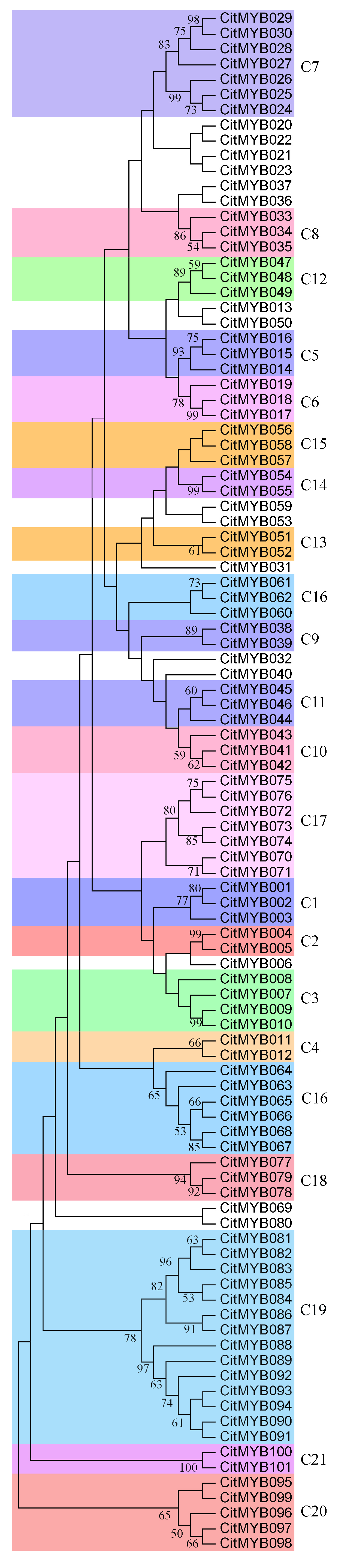

Supplement: Figure S1 — NJ phylogenetic tree of the 101 CitMYB members on the basis of the MYB domain. The bootstrap value less than 50 are not shown in the phylogenetic tree. (TIF) [file pone.0113971.s001.tif]

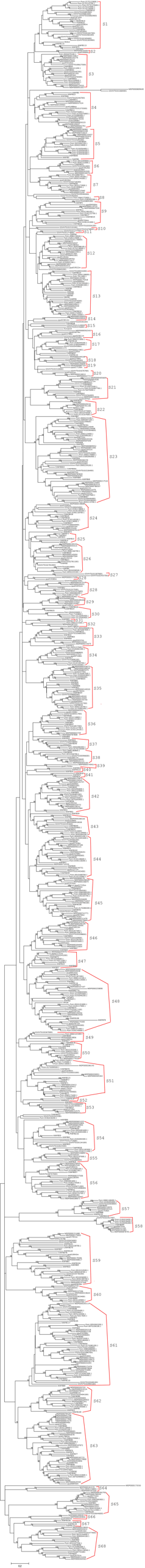

Supplement: Figure S2 — Phylogenetic relationships and subgroup designations in R2R3MYB proteins in Arabidopsis, citrus, apple, peach, populus, grape and other plants. (TIF) [file pone.0113971.s002.tif]
