# Supplementary material for: Genome-Wide Analysis of Citrus R2R3MYB Genes and Their Spatiotemporal Expression under Stresses and Hormone Treatments
Source: PLoS One. 2014 Dec 4;9(12):e113971. doi: 10.1371/journal.pone.0113971 (PMC4256393; doi:10.1371/journal.pone.0113971)
Supplement: Table S1 — Specific primers of 101 citrus R2R3MYB genes used for real-time PCR in this study. The primer based on SAND gene was used as normalizer. (DOC) [file pone.0113971.s003.doc]

Table S1 Specific primers of 101 citrus R2R3MYB genes used for quantitative RT-PCR in this study

| CitMYB001-F | TGATAGCTGGGAGATTGC | CitMYB052-F | TTGTTACCAGCCTCTGAATACC |
| --- | --- | --- | --- |
| CitMYB001-R | TCTGCTTTGTCGATTACCT | CitMYB052-R | CAGCATCGTCTCCTTTCCTG |
| CitMYB002-F | TGGAAGATTACCTGGTCGGA | CitMYB053-F | CTAATGCGACAACTCCAAGC |
| CitMYB002-R | CATCAGCATAAGAAGTTCCCTC | CitMYB053-R | ATCAAGCGACAGCCACTCTG |
| CitMYB003-F | GGGAGACTTCCAGGACGAAC | CitMYB054-F | ATCAACACCATCACCAAACG |
| CitMYB003-R | TGCCACCCTCATCATTTACG | CitMYB054-R | TCGGCAACGAGTCAATCA |
| CitMYB004-F | CAACAACAGCAGCACCTATCA | CitMYB055-F | ATTCATCAGAGCCAAGAGGTG |
| CitMYB004-R | CAACCCTCTTGGACTCATCTT | CitMYB055-R | GTCATAGCGGGTATCAAGCA |
| CitMYB005-F | AATCCAACAACCCACAAG | CitMYB056-F | GGCTGCTGGAGTTCTGTT |
| CitMYB005-R | TGAATCCACCATCAATAGG | CitMYB056-R | GCAAGTGCCTCGCTATCT |
| CitMYB006-F | TCGAGTTCAAGGAACCGAC | CitMYB057-F | TGCTGGAGTTCAGTCCCTAA |
| CitMYB006-R | CCTAATGGGCTTTGGGAG | CitMYB057-R | ATCTGAGCCCACCTGTTTCC |
| CitMYB007-F | TGAAGGAAAATGGAGATACC | CitMYB058-F | TTATTGATAGCCACCCTGACG |
| CitMYB007-R | TTGCCAGGAGATTGAGAA | CitMYB058-R | AGGTTGTTGCTGGTTTTGAG |
| CitMYB008-F | GTTGCCTAAGAGCTTGTGAAGT | CitMYB059-F | CGCATTAGAAGGCGATCAAG |
| CitMYB008-R | TCAAGGCTTATGGTGAAGGA | CitMYB059-R | TGAATCCAAGTGGTGGTGTT |
| CitMYB009-F | CTGAATCTGTATGGTGGGAAAG | CitMYB060-F | GCAAACAGCAACAGGGAATA |
| CitMYB009-R | GGCCTGCTTCAATGACTCTATT | CitMYB060-R | CTGGGAAAGCACTGCAAGTC |
| CitMYB010-F | GCACTTACTGCTCCACCA | CitMYB061-F | TCTATTTCGCCGCCTCAA |
| CitMYB010-R | GACATTGTCCAACTTCACC | CitMYB061-R | TGTGCGTGTCGTGTTCTT |
| CitMYB011-F | AATGTGAACCGAGGCAACT | CitMYB062-F | ACAACCCATTATGGGATTAC |
| CitMYB011-R | GGTCTTTGATCCCCAGCT | CitMYB062-R | AAGGAGGTGTCACAAGCA |
| CitMYB012-F | TCTTGCTTGAGTACATAAGGGTCC | CitMYB063-F | TTACCTGGAAGAACTGACAACG |
| CitMYB012-R | CCACCTGTTTCCAAGGAGATTA | CitMYB063-R | TCACAGGCTTCAGATTTTGG |
| CitMYB013-F | ATGAACCAGCAGTCAAACCC | CitMYB064-F | CTGGATTGCTTTACCCAAGA |
| CitMYB013-R | TCAATTCGGCAGCTCACTAT | CitMYB064-R | AGCTATAACAGACCACCTACTTCC |
| CitMYB014-F | AAAACTCACTACGGCATCCAC | CitMYB065-F | GCAGCCAGATCAAACAAGAA |
| CitMYB014-R | AAAGCCTCTTTTCGCTCGTT | CitMYB065-R | GCAATTTCCCCAAAATACCC |
| CitMYB015-F | ACAACACCTCCACCACCAAC | CitMYB066-F | CGACATCAACGCTTATTACACC |
| CitMYB015-R | TAGAAGGCGCAAGTCCAATG | CitMYB066-R | ACTAGCTTGATCGCCTCCAA |
| CitMYB016-F | CCTCCTTGGTAACAAGTGGTC | CitMYB067-F | GCAAGCAACGCAAAGAACAG |
| CitMYB016-R | GCTACAGGTACTGCTGGCTCA | CitMYB067-R | AAAGGTAGCTCAGGCCAGTAA |
| CitMYB017-F | GCTTGCGTTCCTGCTCTTCT | CitMYB068-F | CAGTTGGATTCACAGCACAGG |
| CitMYB017-R | GTGACTCTTGGGTTCATCCTTATC | CitMYB068-R | GATTGATGAATAGCAGGCGTAG |
| CitMYB018-F | GCAGAAGACGAAGTGAAAAGG | CitMYB069-F | AAGAAACAATGCCAAGCG |
| CitMYB018-R | CCCAGTCAAAATCAAGCCAT | CitMYB069-R | CGATAACTCTGACATCCAATC |
| CitMYB019-F | TGAGGTTTGGACTTTACTGGAG | CitMYB070-F | GCCGATGGCTATGGAGAATC |
| CitMYB019-R | TTACGGAAGAGCAGGCAACA | CitMYB070-R | GCAGTTAGTGCTTGCGTCAA |
| CitMYB020-F | CGGGGCAGACTCATTAACAT | CitMYB071-F | AGATGGGGAAACAGGTGGTC |
| CitMYB020-R | TCGTCTCATCAAATCACCTCC | CitMYB071-R | GGTGCTGTTGTTGCTGATGAA |
| CitMYB021-F | CTCCGGTGGATAAATTACTTGC | CitMYB072-F | AGGCGTCTGGAACTCACT |
| CitMYB021-R | TTGCTTTTGCTTGAGACGCT | CitMYB072-R | TCCTGGTAGATGCTTTGC |
| CitMYB022-F | GAGATGTGGAAAGAGCTGTAGG | CitMYB073-F | TAGACGCAAATAGCACGACA |
| CitMYB022-R | TTGTCTGTTCGTCCTGGTAATC | CitMYB073-R | GCTGATCCCTGATGATGAAA |
| CitMYB023-F | AGATTCGTCTTGCTCTTCGG | CitMYB074-F | CAGCAACCAAATAGTCCCAAAC |
| CitMYB023-R | TGATCTCAGGCTTATTCACCTC | CitMYB074-R | CATCCATGTTCCACAAAGCA |
| CitMYB024-F | TACGGCATTTGGAACTGGAG | CitMYB075-F | GAGTCATGGGGCTGGTCTAA |
| CitMYB024-R | TTTCCAAGTTGTTCATGCAG | CitMYB075-R | CATCTTCTGAAAGCTGAAGTGC |
| CitMYB025-F | TTAGGAGATACGGCATTTGG | CitMYB076-F | TTTCATCTGTCTCGGACTTGG |
| CitMYB025-R | ATGCAATAGCAGACCATCGA | CitMYB076-R | TTATCAGACGCATCTCCACC |
| CitMYB026-F | CTTCCCCACAACTTTCTGCT | CitMYB077-F | CCACTTGATCCCGATACTACTC |
| CitMYB026-R | ATAACCCTCCGCCATAGTCA | CitMYB077-R | TGTTCCCCAGCTACCTAAATC |
| CitMYB027-F | AAGTGACCAGATTTCCTCCTTG | CitMYB078-F | CTGTATCCACCGGAGATT |
| CitMYB027-R | TCAGAATAGAACGGCTCGGT | CitMYB078-R | GATGAAGGGAGTGATGTTG |
| CitMYB028-F | AAATGGTCCTTGATTGCTGC | CitMYB079-F | CGCTCAGTTACCTGGAAG |
| CitMYB028-R | GTCTTTCGCTTTCTGCTTGA | CitMYB079-R | TGGTGATGGAGATATTTGG |
| CitMYB029-F | TGCTGGTCTATCAAGGAGTGG | CitMYB080-F | CAGGCACAGTTTGGGAACAA |
| CitMYB029-R | GTTCTTCCCGGTAGTTTTGC | CitMYB080-R | AAAGTGGAAGCATCGGGAGA |
| CitMYB030-F | TGCTGGTCTATCAAGGAGTGG | CitMYB081-F | ATTGAGTCTGTCCCTTCCTGG |
| CitMYB030-R | GTTCTTCCCGGTAGTTTTGC | CitMYB081-R | CTTCCTGCTTTATCATCTCCTG |
| CitMYB031-F | ACCATCATCATTATCTGCCACC | CitMYB082-F | GAACCAGGTGGCATCAACAC |
| CitMYB031-R | GAATGCTGAGACTCAACCCA | CitMYB082-R | CTTCAGCAGGCAAACAGAGC |
| CitMYB032-F | AAAGAGTTGCAGACTCAGATGG | CitMYB083-F | TTGATGAAGACAGCGAGGAT |
| CitMYB032-R | CTCATTATCCGTTCGTCCAG | CitMYB083-R | AGGCAAGCCCAGAGTAAGAT |
| CitMYB033-F | AATCTCAAGGGGTCAGTGGG | CitMYB084-F | CTCCACCTTTAGTCATTCTTGC |
| CitMYB033-R | TGGTGCTTGAACTGCTGGTT | CitMYB084-R | GGGTCGCTCTTCGAGTTTTA |
| CitMYB034-F | TCATTCACCAACACGGCATA | CitMYB085-F | GACTTTGTTGACGTTGTCGC |
| CitMYB034-R | CTTTCATCCTGAAACAGGCTAG | CitMYB085-R | CCACCGTACACCTGTCCTCT |
| CitMYB035-F | ATAATACTCACCCGTTTGCG | CitMYB086-F | CATCCAAGGCAAACTCCA |
| CitMYB035-R | ATGATCTCATCCAGCCTTCG | CitMYB086-R | ATTCAGGCCCCAACAGAG |
| CitMYB036-F | TCTGGAGGATTGAGCGAAAA | CitMYB087-F | GGGGTGAGTTTACTGCTG |
| CitMYB036-R | GAGCCAATGGAGTCTGAAACAG | CitMYB087-R | GGAAGGGTAGGATGAGGT |
| CitMYB037-F | TCCCTAGATAAATACCGTAGCAGC | CitMYB088-F | CATAGCTGAAAAGCTCCAAGG |
| CitMYB037-R | CACCACCAACAGTAGCACCA | CitMYB088-R | CGTTATCAGTACGTCCAGGAAA |
| CitMYB038-F | GTCAACAAAGTGAATGGCAGAG | CitMYB089-F | GCGGTTCTAGTTCATTTGGG |
| CitMYB038-R | CGACAAAACTGAAGCGAAGT | CitMYB089-R | GATCATCTGCTTGAGCTTGTTG |
| CitMYB039-F | GGGACGTACCGACAATGAAA | CitMYB090-F | AAGGAGATTACGAAGGGAAGTG |
| CitMYB039-R | AGAAGTGACGCCGATGAACC | CitMYB090-R | GGAGTTGTTGTTGCCTACTGACT |
| CitMYB040-F | CATGGAAGATGGAGAACCCTT | CitMYB091-F | CCAGCATTTATAGGAGGAG |
| CitMYB040-R | CTGACCACTTGTTTCCCAAA | CitMYB091-R | TGGAAAGGACCAGAAGAG |
| CitMYB041-F | TGTATTACTAAGTGGACCACCAGC | CitMYB092-F | TCCACAACTGCAACAACC |
| CitMYB041-R | AACAGAACCATCCGTAGCGT | CitMYB092-R | CAAGAAAGTCAATGAACCGT |
| CitMYB042-F | CAAGCGACGGTCAAAACAAG | CitMYB093-F | GAGGACTCAAAGCTCAAGG |
| CitMYB042-R | CAGGAGGCGGAACAGAAGAT | CitMYB093-R | CACTCTATGTGCTGCCATTA |
| CitMYB043-F | CCAGTGTTAATAACGCAGGAG | CitMYB094-F | CAAGCTCCCATTGGTTAC |
| CitMYB043-R | GGTGTCGTATTTGGCTGTCTA | CitMYB094-R | GATAGTTGCTTGAATGTTGC |
| CitMYB044-F | TGTACTCGGAAACAAGTGGTC | CitMYB095-F | TCAACTCTGATCCCTCGTCC |
| CitMYB044-R | CAGCAAGCAACTGAGGCAGA | CitMYB095-R | CACCACCAGAAGTAAATAACCC |
| CitMYB045-F | CACAAGTCCCTTTCAGTTTCC | CitMYB096-F | GGTCTACGGTCATCACATT |
| CitMYB045-R | GCATCACCTGGATTGCTTAC | CitMYB096-R | TCTCCCTCTTCACCTCCT |
| CitMYB046-F | GCTTTTCGCCTAAATCTCCA | CitMYB097-F | TTGGAGTGAGGAGGAAGATAAG |
| CitMYB046-R | GCAGCTACAACTGATCCTCCA | CitMYB097-R | CTCTGGGGTATTTTGAACGA |
| CitMYB047-F | GAGCAGCAGGAGAAACCAAG | CitMYB098-F | GCCAAGCACCACTACTACTACA |
| CitMYB047-R | TGATGACGAAGCTGTAGGGA | CitMYB098-R | TCTTATGCCCAATCATCCCT |
| CitMYB048-F | AGCAAAAGGCGGAAAGTAGT | CitMYB099-F | GCCCACGAATGAATACCAAA |
| CitMYB048-R | GAGGAGGTTCATCAATCCACAA | CitMYB099-R | GTCTAGGAAGCTCCCTCTGC |
| CitMYB049-F | ACAATAAGGAGCCTGAGACATC | CitMYB100-F | TTTCGTTGCAGGCCAAATAC |
| CitMYB049-R | GCTCGATCAAAGGAACATCA | CitMYB100-R | AGAACCCAAACCCACTACCG |
| CitMYB050-F | TTATCTGAGACCGGACCTTAAG | CitMYB100-R | AAACCTCCGTTAGAAGTGGC |
| CitMYB050-R | TCACGGGGTCTAATCCAAGC | CitMYB101-F | TGGTGGGAAGTGTTCAAAGA |
| CitMYB051-F | CAAAGCTGCAACAGCAAAGA | *SAND*-F | ATAATCAAAATCGTCAACATGC |
| CitMYB051-R | ATTGCACCAGCCATGAGTGT | *SAND*-R | AGGTTGTCAGCCTTGTTGGT |
